# Oil Sands Contaminants: Editor’s Response

**DOI:** 10.1289/ehp.1103746R

**Published:** 2011-08-01

**Authors:** Bob Weinhold, Susan M. Booker

**Affiliations:** **Bob Weinhold**, Freelance Science Journalist, Colorado City, Colorado; **Susan M. Booker**, News Editor, *EHP*, Research Triangle Park, North Carolina, E-mail: booker@niehs.nih.gov

The article by Weinhold (2011) offered an overview of potential environmental and health issues related to oil sands operations and was never intended to be nor presented solely as a recapitulation of the Royal Society of Canada (RSC) report (Gosselin et al. 2010). Instead, it presented a discussion of the environmental health information in that report as well as the related significant source documents it reviewed.

The information presented by Weinhold (2011) went beyond the conclusions in the RSC report in order to highlight the data used to reach those conclusions. It also provided information from a number of other sources, which at times conflicted with the RSC’s conclusions even as it agreed with details in the report.

Both the RSC report and other reports document a range of health and environmental concerns in the Alberta oil sands operations area and beyond. Weinhold’s article (2011) reflected that evidence and included numerous qualifying statements stipulating that many unknowns remain. The fledgling evidence, combined with major gaps in existing environmental health science and the fact that very little of the expected oil sands development has occurred, suggest that significant additional adverse effects cannot be ruled out as development expands. Given these facts, it would have been irresponsible journalism for Weinhold to have given oil sands operations an essentially clean bill of health.

The photograph on p. A130 of Weinhold’s article (2011) speaks to the reality that many citizens of Fort Chipewyan continue to be concerned about the possible effects of oil sands activity on their health and are uncertain about why community members are dying from what appear to the survivors to be unusual causes. Although Hrudey may feel this concern is misplaced or unfounded, that opinion does not reflect the feelings of those who live in Fort Chipewyan. The Alberta government’s assertion that more extensive health studies are warranted (Chen 2009) and stated intention to actively pursue such studies (Weinhold 2011) suggest adverse health effects are at least plausible.
